# Cytokine expression patterns predict suppression of vulnerable neural circuits in a mouse model of Alzheimer’s disease

**DOI:** 10.1101/2024.03.17.585383

**Published:** 2024-03-17

**Authors:** Dennis C. Chan, ChaeMin Kim, Rachel Y. Kang, Madison K. Kuhn, Lynne M. Beidler, Nanyin Zhang, Elizabeth A. Proctor

**Affiliations:** 1 Department of Neurosurgery, Penn State College of Medicine, Hershey, PA, USA; 2 Department of Pharmacology, Penn State College of Medicine, Hershey, PA, USA; 3 Department of Biomedical Engineering, Pennsylvania State University, University Park, PA, USA; 4 Center for Neural Engineering, Pennsylvania State University, University Park, PA, USA; 5 Center for Neurotechnology in Mental Health Research, Pennsylvania State University, University Park, PA, USA; 6 Department of Engineering Science & Mechanics, Pennsylvania State University, University Park, PA, USA

## Abstract

Alzheimer’s disease is a neurodegenerative disorder characterized by progressive amyloid plaque accumulation, tau tangle formation, neuroimmune dysregulation, synapse an neuron loss, and changes in neural circuit activation that lead to cognitive decline and dementia. Early molecular and cellular disease-instigating events occur 20 or more years prior to presentation of symptoms, making them difficult to study, and for many years amyloid-β, the aggregating peptide seeding amyloid plaques, was thought to be the toxic factor responsible for cognitive deficit. However, strategies targeting amyloid-β aggregation and deposition have largely failed to produce safe and effective therapies, and amyloid plaque levels poorly correlate with cognitive outcomes. However, a role still exists for amyloid-β in the variation in an individual’s immune response to early, soluble forms of aggregates, and the downstream consequences of this immune response for aberrant cellular behaviors and creation of a detrimental tissue environment that harms neuron health and causes changes in neural circuit activation. Here, we perform functional magnetic resonance imaging of awake, unanesthetized Alzheimer’s disease mice to map changes in functional connectivity over the course of disease progression, in comparison to wild-type littermates. In these same individual animals, we spatiotemporally profile the immune milieu by measuring cytokines, chemokines, and growth factors across various brain regions and over the course of disease progression from pre-pathology through established cognitive deficit. We identify specific signatures of immune activation predicting hyperactivity followed by suppression of intra- and then inter-regional functional connectivity in multiple disease-relevant brain regions, following the pattern of spread of amyloid pathology.

## Introduction

13.8 million people are projected to be afflicted with Alzheimer’s disease (AD) over the next couple decades (1,2, 3). It is a neurodegenerative condition characterized by an entanglement of disease pathologies, and cognitive impairment resulting in a decrease in quality of life (4, 5). Classical hallmarks of Alzheimer’s Disease are the development and spread of neurotoxic amyloid-β peptides and oligomers (6–17) and neurofibrillary tau tangles (13–17) in parallel with neuroinflammation (15–20) and, ultimately, neuronal loss and brain tissue atrophy. The clinical symptomatic aspect of the described pathologies is the loss in cognitive performance, with cognitive impairment manifesting in spatial cognition, working memory and executive functions (16, 21). However, current treatment options for Alzheimer’s Disease have failed to safely and meaningfully delay, slow, or halt disease, let alone reverse the effects on cognition (22, 23), potentially due to focus on single molecular targets. Integration of information from multiple aspects of AD pathology is likely needed to interrogate and correct the complex, systems-level mechanisms negatively impacting neuronal health, brain function, and cognition in AD.

One of the emerging biological factors of interest is the role that inflammation plays in the progression of Alzheimer’s Disease. It has been detailed that neuroinflammation occurs over the progression of Alzheimer’s Disease (15–20). On a more foundational level, it has been shown that neuroinflammation can have detrimental effects on neuronal behavior, such as predisposing neurons to be more sensitive to intrinsic excitability, resulting in increased rates of spontaneous action potentials, leading to an uncoordinated neuro-electrical behavior (27, 28). Moreover, cytokines have also been shown to have a profound effect on synaptic plasticity (28–30), demonstrating that microglia responses to adverse stimuli can affect neuronal behavior. Such effects can manifest in alterations to cognitive performance (28, 31), thus establishing a link between neuroinflammation and cognitive ability.

While the inflammatory environment is one neurophysiological event that influences cognitive behavior, another quintessential factor to driving cognitive performance is the level of neuronal activity (35, 36). This neurophysiological event can be described by brain regional activity and the regional combined behavior during a specific cognitive state. Thus, depending on the experimental paradigm, one can quantify how particular brain regions are involved at specific tasks (32–34), providing insight as to which region are involved in executing a particular task, quantifying what is called a functional network. While task based functional connectivity metrics yield insight into task specific networks, studies focusing on resting state functional connectivity (rsFC) have yielded a wealth of information as to how the brain organizes when no task is being performed (37, 38). It was found that such networks quantified during the resting state were different between comparing healthy controls to diseased populations or subjects afflicted with cognitive impairment (35, 36, 39–41).

Therefore, there is sufficient evidence that the regional inflammatory environment surrounding bodies of neurons can affect their behavior, and thus affect the spontaneous activations of brain regions that could influence cognitive behavior. However, the major limitation is that the majority of studies examining the above-mentioned factors study them in a univariate manner, excluding details how each individual factor affect each other over the progression of Alzheimer’s Disease. Existing efforts to rectify the limitation includes the use deep learning or artificial intelligence to evaluate relationship cross-modality in large datasets (24–26). These datasets contain a wealth of information, and online databases such as the Alzheimer’s Disease Neuroimaging Initiative (ADNI) offer a more comprehensive quantification of disease pathology. However, such datasets lack spatial resolution when it comes to examining the inflammatory environment of individual brain regions, and only provide terminal data when quantifying the inflammatory environment of the brain. The consequence is that one can not determine how the development of Alzheimer’s Disease can affect brain regions, thus driving changes to functional connectivity which then manifest as alterations to cognitive performance.

In our attempt to address the current limitation in the field, we describe an experiment that is centered around a well stereotyped mouse model of Alzheimer’s Disease, and quantify the inflammatory environment of 3 cortical brain regions over four time points that are related to cumulative key events that occur during Alzheimer’s Disease progression; before the development of disease pathology at month 1.5 (42), extracellular amyloid deposition occurring at month 2 (42, 43), deterioration of basal synaptic transmission in hippocampal CA1 at month 4 (42), and neuron loss at month 6 (42, 44–46). Additionally, we utilize awake resting state functional magnetic resonance imaging (rs-fMRI) to quantify the resting state functional connectivity during those four time points to correlate the functional connectivity to regional brain inflammation over disease progression. Our findings provide a new insight into how neuroinflammation and functional connectivity are intertwined in Alzheimer’s Disease, thus highlighting which brain regions and networks are implicated in disease pathology at different stages of the neurodegenerative disease.

## Methods

### Animal subjects.

Institutional and national guidelines for the care and use of laboratory animals were followed and approved by the Penn State Institutional Animal Care and Use Committee (PRAMS201647005). 5xFAD hemizygous B6SJL (MMRRC Strain #034840-JAX) background strain and wildtype mice were used to breed a colony of mice that were either hemizygous for the 5xFAD related genes, or wild-type littermates. All mice were genotyped following the protocol provided by Jackson Labs (Protocol Number: 31769). All mice were also genotyped for the retinal degenerative gene Pde6B using the protocol provided by Jackson Labs (Protocol Number: 31378). Animals that were found to be homozygous for the Pde6B gene were excluded from the study. Both male and female animals are included in the study. Mice were singly housed following headpost surgery (procedure below) at 1 month old. Food and water were provided *ad libitum.* Nesting material and chew blocks were provided to singly-housed mice as enrichment. The housing room was kept at 70 °C and adhered to a 12-hour light and 12-hour dark cycle. The study consisted of 160 mice: 27 for longitudinal imaging studies, 31 for age 1.5 months single timepoint, 33 for age 2 months single timepoint, 34 for age 4 months single timepoint, and 35 for age 6 months single timepoint. All mice underwent rs-fMRI. Cytokine protein levels were profiled in 105 animal brains from the same cohort of animals that underwent rs-fMRI: 25 mice for age 1.5 months, 26 for age 2 months, 27 for age 4 months, and 26 for age 6 months. All experiments were conducted during the 12-hour dark cycle to better capture the properties of the awake state, with euthanasia and tissue collection (procedure below)_directly following imaging. All experiments in the present study were approved by the Institutional Animal Care and Use Committee (IACUC) at the Pennsylvania State University.

### Surgical Procedures.

Mice had headpost implantation surgery done at 1 month old. Mice were anesthetized with 3% isoflurane and an oxygen flow of 1.0 L/min within an isoflurane chamber. The surgical plane of anesthesia was confirmed by pinching the lower limbs of the mouse, and if no reaction was observed, the mouse was successfully anesthetized. The scalp of the mouse is then shaved using an electric clipper in preparation for surgery. A heating mat with a rectal probe (*PhysioSuite* with *RightTemp* from Kent Scientific) is used to maintain a body temperature around 36–37 degrees Celsius. A custom surgical setup that features the boundaries of the awake animal restrainer used in neuroimaging experiments is used. Once the mouse is in position, and the head is fixed for surgery, the scalp is cleaned twice alternating between provodine iodine and 70% isopropyl alcohol. Ophthalmic ointment is applied to the animal’s eyes to prevent dryness during the course of surgery. The scalp is infiltrated with Bupivacaine (4mg/kg), followed by an anterior to posterior incision before excising the skin to form a circular excision anterior to the ears, and posterior to the eyes. All relevant soft tissue is then removed such as the periosteum, leaving only the skull exposed. The site is washed one time with sterilized saline solution. Once dried, an etching solution (composed of citric acid) is applied to the skull for 30 seconds before being rinsed off using saline solution. Dental cement (C&B Metabond) consisting of a ratio of 1 drop catalyst (C&B Metabond), 4 drops of “quick!” base (C&B Metabond), and 2 scoops of clear L powder (C&B Metabond) is applied to the exposed skull using a fine tipped brush. Rough edges are smoothed out during the process. Once set and cured, a custom head post constructed from polylactic acid (product number) is adhered to the dental cap using dental cement of the same recipe. Upon completion of procedure, meloxicam (5 mg/kg) is administered 2 days post-surgery. Weight and behavior in home cage is subsequently monitored for seven days where any weight drop lower than 20% of pre-surgical weight is recorded as a failure to recover from surgical procedure, and thus excluded from subsequent experiments. If weight and behavior is observed to be satisfactory (no weight loss, no abnormal behavior in home cage) for 4 consecutive days post-surgery, the mouse is then moved to subsequent experiments detailed below.

### Acclimation Procedure.

Mice were acclimated for awake animal imaging using a procedure adapted from an in-house pipeline. The mice were habituated to the restrainer used for imaging where the process lasted 4 days prior to the imaging experiment. An acclimation box was used alongside custom made holders that mimic the interior of a scanner. All mice were first anesthetized using 3% isoflurane, and an oxygen flow of 1.0 L/min. Once anesthetized, mice had their front limbs bounded together using tape before restraining the mice by attaching a custom-made head bar that connected the implanted headpost to the custom-made restrainer. Once the animals were confirmed to be awake, they were given five minutes to adjust to being restrained before the acclimation process started. The first day of acclimation was 15 minutes with no sound. The second, third and fourth day of acclimation was 30 minutes, 45 minutes, and 60 minutes respectively. A soundtrack that had the recording of the noise heard during an fMRI session was played for the stated durations at the second, third and fourth day at 110 db. Once acclimation was over, the mice were anesthetized before being removed from the restrainer, and then placed back into their home cage. Weights before and after restraining were recorded, alongside any behavioral abnormalities such as tears forming.

### fMRI Imaging.

Awake resting state imaging was conducted on a 7T MRI system interfaced with a Bruker Console (Billerica, MA) housed at the High Field MRI Facility at Pennsylvania State University, University Park. Gradient Echo images were acquired using an echo-planar imaging (EPI) sequence with the following parameters: repetition time (TR) of 1.5 second, echo time (TE) of 15 milliseconds, flip angle = 60°, matrix size = 64 × 64, FOV = 1.6 × 1.6 cm^2^ number of slices = 16, slice dimensions = 0.25 mm × 0.25 mm × 0.75 mm. 10 or 32 dummy scans were taken before each EPI acquisition. A trigger delay of 10 milliseconds was set at the beginning of each volume acquisition such that real time monitoring of respiration rate can be synchronized to the volume acquisition. Respiration was monitored using a custom-made nose cone perforated with angled holes to allow carbon dioxide to diffuse out and oxygen to diffuse in without significant loss of pressure. The respiration monitoring system was connected to the 7T MRI system using a MR-compatible monitoring and gating system (Model 1030 from Small Animal Instruments Inc.), which was also interfaced with a desktop that housed the respiration monitoring software from Small Animal Instruments Inc. A total of 4 or 5 EPI scans were collected for every imaging session.

Following the completion of EPI acquisition, a structural T1 RARE image was collected. The imaging parameters are as follows: repetition time (TR) of 1500 seconds, echo time (TE) of 8 milliseconds, flip angle = 90°, matrix size = 256 × 256, FOV = 1.6 × 1.6 cm^2^ number of slices = 16, slice dimensions = 0.25 mm × 0.25 mm × 0.75 mm.

### fMRI Image Preprocessing.

fMRI images and the corresponding T1RARE structural images were preprocessed following a paradigm adapted from an in-house pipeline (47, 48). All processes and scripts described in this section were done using MATLAB 2022b. Briefly described, EPI time courses were first converted from the standard Bruker file format to a .sdt file format before undergoing motion scrubbing. The following formula was used to determine the degree of motion for an isotropic shape:

$$Motion\;Parameter\;=\left|T_{z}\right|+\left|T_{y}\right|+\left|T_{x}\right|+\left|\theta_{x}r_{z}\right|+\left|\theta_{y}r_{z}\right|+\left|\theta_{z}r_{x}\right|$$

Euler angles were back computed from the affine transformation matrix calculated using an internal MATLAB function. Volumes that exceeded half a voxel size of displacement (0.125 mm) were discarded, along with one volume preceding it, and one volume after the volume exhibiting excessive motion. Scans that had more than 10% of volumes discarded due to motion (including the number of neighboring volumes) were discarded. Thus, scans with at least 90% remaining volumes post motion scrubbing were kept for following preprocessing.

Post motion scrubbing, each scan will undergo the process of co-registration and normalization. First, the first frame for each EPI scan is co registered to the relevant subject’s T1RARE structural image using an in-house GUI made in MATLAB. Following co-registration, the subjects T1RARE image is then normalized to a reference brain atlas from the Allen Brain Atlas (WEBSITE). After successful normalization, the affine transformation matrix from the normalization process is then applied to the co-registered EPI images to align the EPI images to the reference brain atlas. Once the first frame for every EPI scan has been successfully aligned to the reference template following the beforementioned procedure, the scans are then motion corrected using functions from Statistical Parametric Mapping (SPM12, 49). Post motion correction, a custom mask for every EPI scan was manually drawn using the first frame for every motion corrected scan. The custom mask excludes areas with significant signal distortion, and is subsequently applied to the postprocessing step. White matter and cerebral spinal fluid (WM-CSF) masks are constructed using the reference atlas to determine the spatial location of the specified features. The signal in the WM-CSF mask is then used as a nuisance regressor alongside the motion parameters calculated from motion correction using SPM12 in a general linear model. The residuals from the general linear model is then spatially smoothed using a 3×3 Gaussian kernel, followed by temporal filtering using a bandpass filter with a cutoff frequency of 0.01 Hz and 0.1 Hz. The end result is a correlation matrix consisting of pairwise correlations between each ROI in the mouse’s brain.

### fMRI Image Postprocessing.

fMRI ROIs were grouped into seven anatomical systems for system ROI analysis. These seven systems were the Sensorimotor Cortex, Heteromodal Cortex, Olfactory Cortex, Hippocampus, Striatum-Pallidum, Thalamus, and Hypothalamus. System representative functional connectivity values are calculated as the mean correlation value where all related ROIs correlation values first undergo fisher Z transformation from r space to z space, before calculating the mean, and then back transformed into r space. The process is repeated for every scan within a group, and the group average are also quantified in the same manner.

The initial evaluation of the entire dataset comprised of examining the correlation matrices in a univariate manner, where every pairwise functional connectivity value is fisher-z transformed (using the arctanh function), followed by implementing a linear mixed effect model of the following structure:

y=Xβ+Zu+ε

Where y is the response vector with nx1 dimensions, n being the number of subjects. X is defined as the predictor matrix, with n×p dimensions, where p is the number of fixed effect predictors (dependent on analysis), β is the p×1 vector of fixed effect coefficients. Z is a n×q⋅m matrix where there are q random effects and m groups. u is defined as a random vector with dimensions q⋅m×1 for m groups. ε is the residual vector for n groups. The random effects are the subjects While the fixed effects are some combination of genotype and timepoint, dependent on the statistical question being evaluated (e.g. time point segregated data will only have genotype as the fixed effect to evaluate differences at a single time point). A two-way ANOVA or two-tailed t-test (depending on the model) is conducted to evaluate the statistical significance of the coefficients derived from the linear mixed effect model.

### Anatomical System definitions.

Anatomical systems such as the Sensorimotor Cortex, Heteromodal Cortex, Olfactory Cortex, Hippocampal Region, Basal Ganglia, Thalamus, Hypothalamus consists of sub ROIs featured in the respective systems. The quantification of the inter and intra system connectivity is achieved by calculating the mean of the fisher z’s transformed correlation values of all ROIs within a system-system interaction. For example, if we are to calculate the system connectivity between the Hippocampal region and the Heteromodal Cortex, then all functional connectivity values between the ROIs within both systems are averaged following the steps described previously. The system definitions for each individual ROI is listed in a table in the supplementary materials.

### Brain Extraction.

Mice were euthanized via decapitation by placing the awake mouse into a decapicone and using a guillotine. The decapitated head was then sprayed with 70% sterile ethanol. The skin and muscle are removed from the skull by removing the skin surrounding the cement cap. Forceps are used to grasp the head post attachment and apply a measured amount of force to slowly rock the dental cap back and forth to slowly loosen the cement cap from the skull. Once the cement cap is ‘peeled’ off from the skull, dissection scissors are used to cut up the side of the spinal cord and around the base of the skull at the lateral and ventral borders. The scissors are then inserted at the base of the skull and are used to make a cut down the midline of the skull from posterior to anterior direction. The dorsal part of the skull is then removed and the brain is slowly and carefully dislodged and placed in a separate cell culture dish containing chilled dissection media (x mL of HBSS and x/90 mL 1 M HEPES) hosted within a bed of ice and placed on top of a chilled copper plate.

The brain is then halved into two separate hemispheres, with the right hemisphere to be dissected into individual brain regions. The brain regions are the Cerebellum, Hypothalamus, Thalamus, Striatum, Hippocampus, Occipital Lobe, Temporal Lobe, Parietal Lobe, and Frontal Lobe. Each individual brain region is placed into their own respective centrifuge tubes that holds a mixture of lysis buffer and protease inhibitor. The brain regions are homogenized by mechanical titration before sitting for 20 minutes on ice before being centrifuged at 5000 rpm for 5 minutes at 4 degrees Celsius. The supernatant is then placed into a separate tube, and flash frozen in liquid nitrogen and stored at a −80 degrees Celsius freezer until further use.

### Quantification of protein.

Total protein content in the supernatant was quantified using the Pierce BCA Protein Assay kit (Fisher 23225). Manufacturer’s instructions were followed accordingly. Samples were run using technical duplicates and absorbance was quantified using a *SpectraMax* i3 minimax 300 imaging cytometer (Molecular Devices). Linear regression was used to quantify protein concentration.

### Multiplex Assay.

Cytokine concentrations of dissected homogenized brain regions from 5xFAD mice were quantified using the Bio-Plex Pro Mouse Cytokines Grp1 Panel 23 Plex (Cat. #M60009RDPD) using the Luminex FLEXMAP3D platform. Manufacturer’s protocol was performed with minor modifications to accommodate the use of a 384 well plate. Following steps are applied using existing in-lab protocols (58). Streptavidin-phycoerythin was used at half volume While magnetic beads and antibody solutions were diluted 1:1 and used at half volume. Equal parts of lysis buffer lysis assay buffer were used in the preparation of standards and blanks.

### Cytokine Data Cleaning.

The Xponent software provided by the Luminex System was used to interpolate sample cytokine concentrations using standard curves derived from using the 5 point logistic regression model. Concentrations below or above the standard limit are either set to 0 pg/mL or the maximum concentration on the curve respectively. An in-house pipeline was used to clean the cytokine data (64): https://github.com/elizabethproctor/Luminex-Data-Cleaning (version 1.05). All cytokines with above-background values for at least half of the subjects were used in further analysis of the cytokine data; we included cytokines with fewer non-zero values if the non-zeros appeared biased toward a particular group.

### Partial Least Squares Modeling.

We utilized a linear, supervised multivariate regression model known as Partial Least Squares (PLS) (61,62) to model Cytokine Signatures of disease and healthy control states. The ROPLS package in R (63) was used to run both Partial Least Squares Regression (PLSR), and Partial Least Squares Discriminant Analysis (PLS-DA). PLSR was used to construct a predictive model to model a continuous response variable, such as the timepoint of our covariates. PLS-DA was used to create a classification model that sought to discriminate disease from healthy control cytokine signature profiles. All cytokine data was z scored to mean center the data and have the sample distribution exhibit unit variance.

Random sub sampling cross validation tests were conducted to select the appropriate number of latent variables (LVs) for both PLSR and PLS-DA models. Test sets and training sets were determined by randomly sampling the parent dataset. The number of k-folds, consequentially determining the size of the training and test sets, was determined by the following criteria (64): k-Folds = 3 if the number of samples exceeded 30, or k-folds = 5 if the number of samples building model was less than 30. K-fold cross validation was conducted a hundred times. 3 models consisting of either 1, 2, or 3 LVs were constructed in every iteration of the Cross-Validation process. All models were then used to predict the response variable, or class identity to quantify the prediction accuracy for PLS-DA, or the root mean squared error of cross validation (RMSECV) for PLSR. RMSECV has the following formulae:

$$RMSECV=\sqrt{\frac{\sum_{j=1}^{n}\;\;{\left(x_{j}\;-\;{\overset{ˆ}{x}}_{j}\right)}^{2}}{n}}$$

Where $x_{j}$ is the predicted value for the j^th^ sample, ${\overset{ˆ}{x}}_{j}$ is the actual value for the j^th^ sample, and n is the number of samples in the test set. Every iteration resulted in a completely new random sampling of the parent dataset to produce both the test and training datasets for cross validation. The number of LVs was determined by selecting the models, and their number of LVs that they represented, that had the lowest RMSECV, or highest predictive accuracy. This model is then used for subsequent analysis and significance testing.

Significance testing of the model was conducted by permutation testing. 1000 permutations were done to construct a null distribution of random models, where each iteration involved the scrambling of the response variable of interest with respect to the covariates. The final model’s predictive accuracy or RMSERCV is then used to calculate a z score:

$$Z=\frac{x_{\text{Model}\;}\;-\;\mu_{\text{Null}\;}}{\sigma_{\text{Null}\;}}$$

Where $x_{\text{Model}}$ is the model’s predictive accuracy or RMSECV, and $\mu_{\text{Null}}$ is the mean predictive/RMSERCV of the null distribution, and $\sigma_{\text{Null}}$ is the standard deviation of the null distribution simulated by the permutation process. We then calculate the corresponding p value for the significance of the model by comparing the calculated Z score to the Z distribution.

Models that were determined to only have one latent variable will have score plots that show two latent variables for ease of visualization. However, only the one latent variable model is used to inspect the loadings, and to make interpretations about the cytokine profile with relation to disease state. The models here are also orthogonalized for improved interpretability. The orthogonalization process results in projecting the maximal amount of covariance in the response vector and the covariate matrix onto the first latent variable (65). The variable importance in projection (VIP) score was used to threshold which cytokines drove the separation between classes with respect to the response of interest (66). Thus, loadings from the first latent variable for cytokines that had a VIP score greater than 1 are designated as cytokines of interest, as they are implied to have a greater than average contribution to the model.

### Graph Theoretical analysis.

Every functional connectivity matrix (the preprocessed correlation matrix) is converted into a weighted adjacency matrix where the absolute value of the correlation values is used as the weights of each edge. All graph organizational metrics were calculated using the Brain Connectivity Toolbox (50). Hub regions were identified by allocating a score of 0–4 based on the following criteria: 20% highest strength, 20% highest betweenness centrality, 20% lowest path length, and 20% lowest clustering coefficient (51). The graph theory metrics used to determine the hub score were quantified on the group average correlation matrix for every group. The mathematical definitions for the four topological features are described below:

Strength (50, 52):

$$s_{i}=\sum_{j\in N}\;w_{ij}$$

Where $S_{i}$ is defined as the strength for the i^th^ node. $W_{I,j}$ is defined as the weight of the edge between node i and its j^th^ neighbor, and N is the set of all nodes. Strength quantifies the degree of connection between a node and its neighbors.

Clustering Coefficient (50, 53):

$$C_{i}=\frac{1}{k_{i}\left(k_{i}\;-\;1\right)}\sum_{j,k\in N}\;{\left(w_{i,j}w_{j,k}w_{k,i}\right)}^{1/3}$$

$W_{i,j}$ is the weight between node i and node j, and $k_{i}$ is the number of neighbors of a vertex. The clustering coefficient can be interpreted as the degree to which nodes tend to cluster together. For the local clustering coefficient, $C_{i}$, the coefficient quantifies how close the neighbors are to node i to being a clique. N is the set of all nodes.

Characteristic Path length (50, 55):

$$L^{w}=\frac{1}{n}\sum_{i\in N}\;\frac{\sum_{j\in N,i\neq j}\;\;d_{ij}^{w}}{n\;-\;1}$$

N is the set of all nodes, n is the total number of nodes, and $d_{ij}^{w}$ is the weighted shortest path between nodes i and j. Characteristic path length quantifies the proximity with regards to strength of connection between nodes.

Global Efficiency (50, 56):

$$E^{w}=\frac{1}{n}\sum_{i\in N}\;\frac{\sum_{j\in N,i\neq j}\;\;d_{ij}^{w^{-1}}}{n\;-\;1}$$

N is the set of all nodes, n is the total number of nodes, and $d_{ij}^{w}$ is the weighted shortest path between nodes i and j. Efficiency is essentially the mean of all reciprocals of the weighted distances in a network. The metric quantifies how efficient information is exchanged within a network.

Assortativity (50, 57):

$$r^{w}=\frac{l^{-1}\sum_{(i,j)\in L}\;\;w_{ij}k_{i}^{w}k_{j}^{w}\;-\;{\left[l^{-1}\sum_{(i,j)\in L}\;\;\frac{1}{2}w_{ij}(k_{i}^{w}\;+\;k_{j}^{w})\right]}^{2}}{l^{-1}\sum_{(i,j)\in L}\;\;\frac{1}{2}w_{ij}(k_{i}^{w^{2}}\;+\;k_{j}^{w^{2}})\;-\;{\left[l^{-1}\sum_{(i,j)\in L}\;\;\frac{1}{2}w_{ij}(k_{i}^{w}\;+\;k_{j}^{w})\right]}^{2}}$$

Where $k_{i}^{w}$ and $k_{j}^{w}$ are the weighted degrees of nodes i and $j.\;w_{ij}$ is the weight of the edge between nodes i and j,l is the total number of edges, While L is the set of all edges within the network. Assortativity is used to determine the degree to which nodes connect to other nodes with similar properties within the network.

## Results

### Hippocampal functional connectivity exhibits local suppression before propagating to inter- and intra-regional connectivity loss in thalamus and cortical regions.

**Figure 1.**
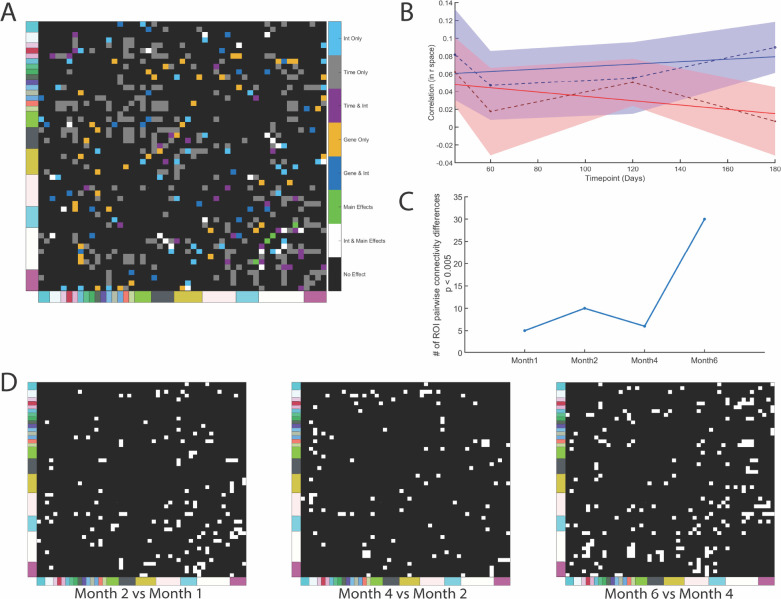
Disease-relevant changes in connectivity of anatomically defined ROIs accumulate non-linearly over time. A) Color-coded binary plot illustrating which connections between ROIs exhibit a disease effect (linear mixed effect model, disease and time as fixed effects, subject as random effect). Interaction term is included. B) Representative time-course ROI-ROI functional connectivity (Postsubiculum and Taenia Tecta), where mean response is plotted with 95% CI. C) Number of ROI-ROI functional connections significantly changed in disease vs. wild-type at each time point, one-tailed t-test with cutoff p < 0.005. D) Binary masks identifying ROI-ROI functional connections significantly changed in disease vs. wild-type at each time point, one tailed t-test with cutoff p < 0.05.

To investigate the effects of Alzheimer’s Disease progression on functional networks, we quantified functional connectivity matrices at four different time points: month 1.5, month 2, month 4 and month 6; utilizing our awake resting state animal imaging paradigm. What we initially observed was that the majority of pairwise ROIs exhibited a time effect, but not an interaction between genotype and time (Figure 1A). We noticed that when examining the connectivity values at each timepoint, the values diverged at later time points, but were parallel to each other at the earlier stages of disease development (Figure 1B), with the divergence in connectivity is markedly shown at the latest stage of disease development, but not exhibited in the other three timepoints. Thus, we proceeded to investigate differences between disease and wildtype mice at each particular timepoint, acting on the assumption that the differences in connectivity values did not follow a linear pattern. The connectivity values were found to be statistically different between disease and control (p < 0.05, uncorrected for multiple comparisons) vary when transitioning from consecutive timepoints and modeling pairwise ROI differences between them for both 5xFAD and wildtype mice in a two way ANOVA model (Figure 1C,D).

**Figure 2.**
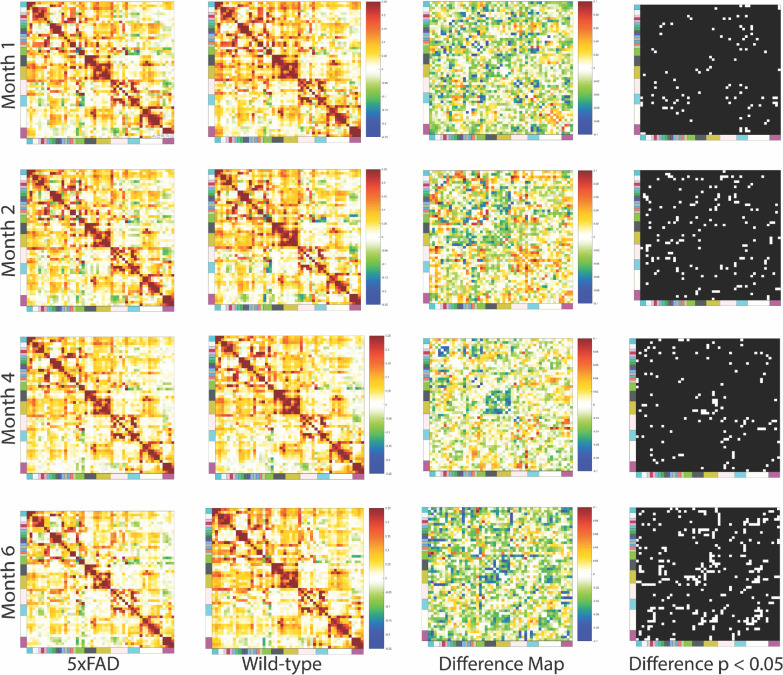
Functional connectivity changes over the course of AD and healthy aging. Symmetric heat maps of Pearson’s correlation coefficients between brain regions of interest. Left to right, column 1: 5xFAD transgenic Alzheimer’s disease mice at age 1.5, 2, 4, or 6 months (rows 1–4, respectively). Column 2: wild-type littermates at the same ages. Column 3: difference matrix representing the changes due to disease (5xFAD minus WT, Z-transformed subtraction). Column 4: binary matrix highlighting connectivity values with two-tailed t test p < 0.05 of LME fixed effect coefficients.

When examining the difference in the global network at each timepoint, we note a couple distinct features of the difference matrices. At month 4 we observe a distinct local depression of functional connectivity in ROIs located in the hippocampal region, and that at month 6 there is a significant increase in the number of pairwise ROI connectivity relationships that are different between the disease and control groups (Figure 2). The observation is further confirmed when examining the binary matrix which highlights regions that exhibited a statistically significant difference (p < 0.05, uncorrected) between the disease and control group. It can be seen that hippocampal ROIs such as the Subiculum, Presubiculum, Field CA1, Field CA2, Dentate Gyrus (DG) and the Entorhinal area exhibit significantly depressed intra-regional connectivity (p_Presubiculum-Field CA2_ = 0.0031, p_Presubiculum-Field CA1_= 0.015, p_Field CA3-Field CA2_ = 0.029, p_Presubiculum-DG_ = 0.041, p_Subiculum-DG_ = 0.041, p_Subiculum-Presubiculum_ = 0.041, p_Presubiculum-Entorhinal area_ = 0.046) at month 4 for the disease group. Some of them such as the retrohippocampal ROIs exhibit lower intra-ROI connectivity While the hippocampal formation ROIs displayed lower inter-ROI connectivity with retrohippocampal regions such as the Presubiculum.

Another feature that we observe is the level of thalamic ROIs that exhibit differences between the two groups at each specific timepoint markedly increases at month 6. Both inter and intra region connectivity for the Thalamus was depressed at the fully symptomatic timepoint (Figure 2). One interesting result is that the 5xFAD group exhibited a higher level of thalamus to Pallidum connectivity relative to the wildtype group at month 2, where both the Globus Pallidus and the Substantia innominate displayed stronger connectivity with subregions in the dorsal Thalamus (p_Midline group of the dorsal thalamus-Globus pallidus_ = 0.038, p_Midline group of the dorsal thalamus-Substantia innominate_ = 0.036, p_Reticular nucleus of the thalamus-Striatum-like amygdalar nuclei_ = 0.028) . This particular feature is lost at month 4, and instead we note that inter-striatum-thalamic connectivity and inter striatum-hypothalamic connectivity are higher for diseased aging at month 4 (p_Hypothalamic lateral zone-Striatum-like amygdalar nuclei_ = 0.021, p_Hypothalamic lateral zone-Fundus of striatum_ = 0.019, p_Midline group of the dorsal thalamus-Striatum-like amygdalar nuclei_ = 0.007, p_Anterior group of the dorsal thalamus-Striatum-like amygdalar nuclei_ = 8.64 e-2)(Figure 2).

All segregated datasets and the resultant statistics had a linear mixed effect model to estimate the coefficients of disease. Thus, the fixed effect is genotype, and the random effect are the subjects. Then a two-tailed t test is run to test the significance of the estimated coefficient. All statistics in this section was not corrected for multiple comparisons.

### Thalamus and hypothalamus systems exhibit early-disease hyperconnectivity with other brain anatomical systems followed by suppression at later disease stages.

**Figure 3.**
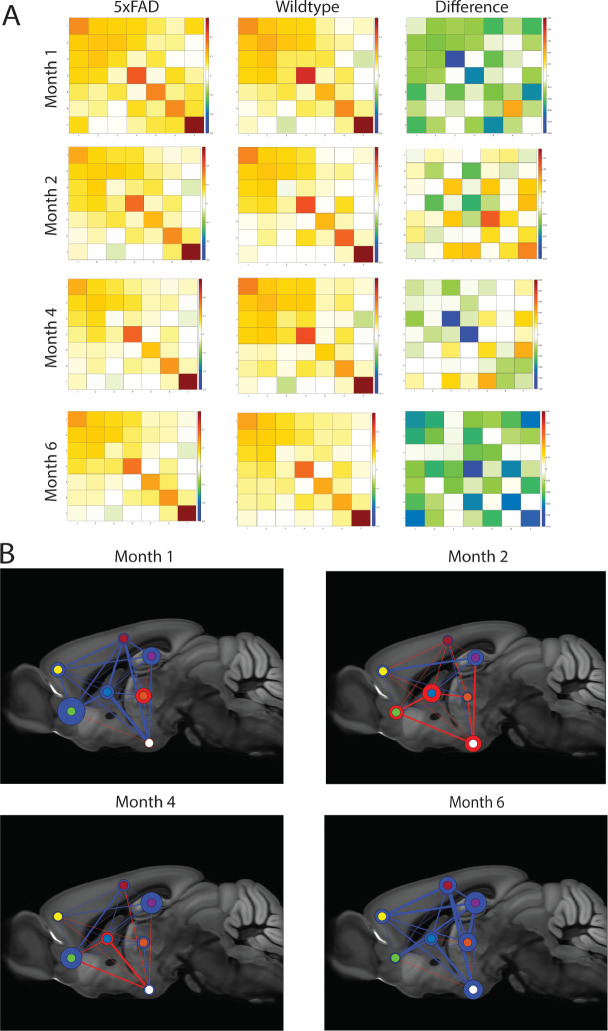
Brain anatomical systems exhibit hyperconnectivity followed by suppression in an AD-relevant spatiotemporal pattern. A) Connectivity matrices and the difference matrix at each respective timepoint, as in Figure 2. B) Spatial illustration of difference matrix in a binarized form superimposed over an anatomical brain slice. Blue: negative change in connectivity, Red: positive change in connectivity. Thicker edges have higher magnitude of change. Color of node outline indicates directionality of intra-system connectivity changes, with greater outline thickness indicating greater magnitude of intra-region connectivity change. Node color: yellow = sensorimotor cortex, red = heteromodal cortex, purple = hippocampus, blue = basal ganglia, orange = thalamus, green = olfactory cortex, white = hypothalamus.

We grouped ROIs and their relevant functional connectivity values into anatomical systems in order to evaluate the effect of disease progression on inter and intra system connectivity. One feature to note is that month 1.5 and month 2 exhibit different anatomical network profiles even though both timepoints are associated with the early stage of disease development (Figure 3A). There is cerebral accumulation of Amyloid plaques that at months 1.5 and 2 (42, 43). However, the difference in concentration between the two points is steep and so month 2 can be used as a proxy to describe a later stage in disease development (43) relative to month 1.5. This feature is reflected in how markedly different the two anatomical networks are, where there is a clear difference in the number of depressed anatomical connectivity values for 5xFAD versus the control (Figure 3A,B). Once again, we note that the thalamic-hypothalamic region in month 2 exhibits a net positive connectivity value in the disease group even though the difference was not found to be significant (p = 0.2570 uncorrected). The systems at month 1.5, however, exhibit all intra-system functional connectivity, excluding the thalamic-thalamic connectivity, to be depressed for the 5xFAD class (Figure 3A,B). All inter and intra connectivity values were found to statistically insignificant (p > 0.05 when corrected for multiple comparisons), and only a handful were shown to be different between the disease and control group at both time points: striatum pallidum-hypothalamus (p = 0.0131 uncorrected, p = 0.1726 FDR corrected) for month 1.5, and intra striatum pallidum connectivity ( p= 0.0243 uncorrected, p = 0.1609 FDR corrected) and Hippocampal-Heteromodal Cortex for month 2 (p = 0.0494 uncorrected, p =0.1635 FDR corrected). The lack of statistical significance when controlling for time falls in line with the projected timeline of disease pathology, where the early stages of disease development may not manifest significant changes to the anatomical networks. However, it must be stated that even though the differences between the two groups in each time point lacked significance, the average anatomical system network profiles were different, and strongly suggests that the increase in amyloid beta plaques could have had a role in influencing the net positive connectivity values for the disease group over the control group at month 2.

At month 4 we observe that the inter-system connectivity of the olfactory cortex and hippocampal region for the 5xFAD animal line is depressed relative to that of wildtype controls (p = 0.0127 uncorrected, p = 0.3545 FDR corrected) (Figure 3 A,B). What we also observed is that the depressed intra-hippocampal connectivity persists at month 6 (p = 0.0011 uncorrected, p = 0.0164 FDR corrected), and that a greater number of systems showed depressed intra-system connectivity at the later stage of disease, such as the sensorimotor cortex (p = 0.0735 uncorrected, p = 0.0978 FDR corrected), Hetermodal Cortex (p = 0.0656 uncorrected, p = 0.0961 FDR corrected), and Thalamus (p = 0.01 uncorrected, p = 0.0487 FDR corrected) (figure 3 A) There were a number of inter-system connectivity patterns that was implicated at the latest stage of disease, such as the Hypothalamus-Sensorimotor Cortex (p = 0.0287 uncorrected, p = 0.06 FDR corrected), Thalamus-Sensorimotor Cortex (p = 0.0209 uncorrected, p = 0.059 FDR corrected), Hypothalamus-Striatum Pallidum system (p = 0.0474 uncorrected, p =0.0771 FDR corrected, and Thalamus-Hippocampal connectivity (p = 0.0040 uncorrected, p = 0.0292 FDR corrected).

Statistics were conducted using a linear mixed effect model on timepoint segregated datasets. Thus, the fixed effect for each model is the genotype, and the random effect are the subjects. Thus, the estimated coefficients that describe the effect of disease on the response is statistically interrogated by a two-tailed t test.

### Global brain networks become weaker, less integrated, and less efficient with disease progression.

Given that prior analysis demonstrated that the ROIs implicated over disease progression did not remain consistent throughout the timepoints, and that the system level analysis yielded a similar perspective, we wanted to investigate how these different network profiles at different timepoints affected network integration and segregation. We utilized the Brain Connectivity Toolbox (50) to quantify metrics related to network integration and segregation where metrics such as global network clustering and assortativity indicating the degree of segregation, and shortest path and efficiency demonstrate integration. We first investigated how disease progression would affect network segregation and integration by first treating timepoints as a continuous variable and running a two-way ANCOVA. None of the interaction terms between genotype and timepoints were found to be statistically significant (p_strength_ = 0.19458, p_charPath_ = 0.2023, p_clustering_ = 0.19342, p_efficiency_ = 0.19037, p_assortativity_ = 0.64371). However, given what we had observed in prior analysis regarding how the functional connectivity values did not follow a linear pattern over time, we then proceeded to investigate the degree to which disease state was correlated to changes in global network properties by treating each timepoint as a categorical class. The following analysis was conducted using a two-way ANOVA with genotype and timepoint as the factors.

**Figure 4.**
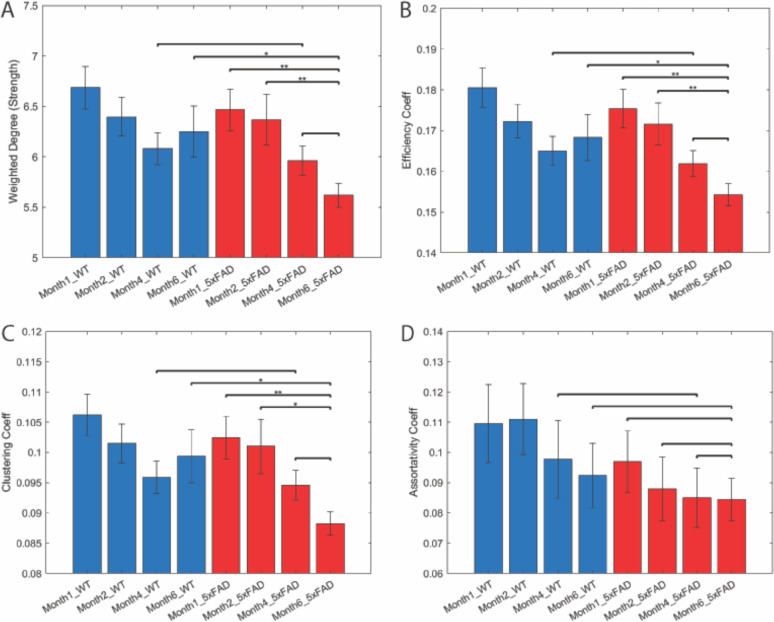
Global brain network is broadly negatively impacted by AD progression. Strength (A), efficiency (B), clustering coefficient (C), and assortativity (D) of the global brain network in transgenic AD (red) and wild-type littermate (blue) mice at the indicated ages. Error bars represent the standard error of the mean. Comparisons by two-tailed t test, *p< 0.05, **p< 0.005.

Global network strength is shown to have differences at particular aging timepoints. The fully symptomatic state (timepoint month 6) exhibited a significantly lower degree of network strength than the early stages of disease development (p_5xFAD:M6 – 5xFAD: M1.5_ = 0.005 and p_5xFAD:M6 – 5xFAD:M2_ = 0.012), and that the disease development had a distinct effect on network strength when compared to healthy aging at the terminal time point (p_5xFAD:M6 – WT:M6_ = 0.037) (Figure 4A). On the other hand, we did not observe any significant differences in network strength in healthy aging, and we note that network strength was not found to be different at the later stages of disease development (p_5xFAD:M6 – 5xFAD:M4_ = 0.252). Another metric that evaluates network integration is global efficiency, which is quantified as the inverse of the global characteristic path length between all nodes in the network (59). We observe the same pattern regarding global efficiency as global network strength. Healthy aged animals exhibited a different global level of efficiency than fully developed symptomatic mice (p_5xFAD:M6-WT:M6_ = 0.036) (Figure 4B). What was surprising was that when controlling for age, we do not notice any differences in the networks efficiency until we compare fully aged animals against fully symptomatic animals, as such the intermediate state of disease development at month 4 exhibited insignificant differences at the efficiency metric relative to a healthy animal of the same age (p_5xFAD:M4-WT:M4_ = 0.630).

Network integration metrics illustrate how well connected the nodes of the network are, and as a result one can infer how efficient information can be transferred across nodes, and what the ease of communication is between nodes (59, 67, 69). On the other hand, functional segregation allows one to infer how specialized certain regions of a network are. Such metrics can quantify groups and communities, some of which may exhibit special properties or functions depending on the context (59, 67, 68, 69). Therefore, we’ve quantified a metric of network segregation: clustering coefficient. Just like the global metrics of network integration, we observe similar trends regarding which age features differences between diseased and healthy animals (Figure 4C). Fully symptomatic animals had a lower clustering coefficient than that of healthy aged animals (p_5xFAD:M6-WT:M6_ = 0.033), and that the later stages of disease development resulted in a lower clustering coefficient compared to early states of disease progression (p_5xFAD:M6–5xFAD: M1.5_ = 0.007, p_5xFAD:M6–5xFAD: M2_ = 0.013). Once again, the differences between the two groups is not observed at month 4 (p_5xFAD:M6-WT:M4_ = 0.799).

Another metric of interest is assortativty. Assortativity can be interpreted as a metric that defines how one node exhibiting a set of characteristics tend to connect with other nodes with the same features (59, 69). Surprisingly, we do not observe any statistical differences between any groups and their respective timepoints. One interesting feature to note though is that at the beginning stage of disease development, we note that there is an appreciable difference in the Assortativity coefficient at month 1.5 (p_5xFAD:M1.5-WT:M1.5_ = 0.421) (Figure 4D).

Statistics were conducted by using a two-tailed t test to interrogate if any significant difference exists between two metrics of interest (i.e. month 6 control versus month 6 wildtype). All graph theory metrics were derived using the Brain Connectivity Toolbox (50), and the graph metrics were quantified on every scan for every subject, before a subject average is quantified. Then the subjects’ averages are pooled together to quantify an arithmetic mean.

### Distinct regional cytokine signatures predict disease progression.

There are regional differences regarding which cytokines drive aging for both diseased and healthy groups. We identify key cytokines driving aging in both disease and healthy groups separately, and have mapped a consensus cytokine list between the two groups per brain region based on cytokines that had a VIP score greater than 1 for at least one of the two groups (disease versus healthy). From there, we examined how different the loadings are for these cytokines of interest.

**Figure 5.**
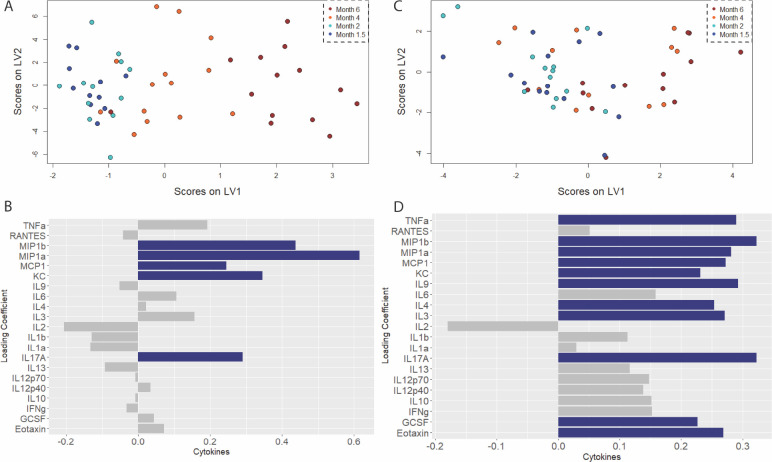
Parietal cortex exhibits progressive and specific up-regulation of microglial activation signature coinciding with onset of synaptic dysfunction. A) Scores plot and B) loadings on the first latent variable of an orthogonalized partial least squares regression model of cytokine levels in the parietal cortex regressed against age as a measurement of disease progression. C) Scores plot and D) loadings plot for the companion model in wild-type littermates. Colored bars indicate cytokines with VIP score > 1.

For the parietal cortex, we identify key cytokines upregulated in our disease model (3 LVs, p = 1.939 e-5, RMSECV = 22.201, Figure 5A,B) such as MIP-1α, MIP-1β and CXCL-1 (also known as keratinocyte-derived cytokine; KC). Thus, there is an upregulation of cytokines canonically associated with having a pro-inflammatory role (71, 72, 73, 74, 75) for diseased aging in the Parietal Cortex. However, in the healthy aging group (1 LV, p = 9.399 e-5, RMSECV = 43.202, figure 5C), we note that there is an upregulation of cytokines canonically associated with immune regulation such as MCP-1, GCSF, IL-4 and IL-9, and may exhibit neuroprotective roles (76, 77, 78, 79, 80, 84) in the brain. One key note is that these ‘neuroprotective’ cytokines are not seen as a covariate that drives aging in the disease group, but are in the healthy group. Another feature is that MIP-1α is significantly more upregulated in the disease group compared to the control group. It is observed that cytokines predominantly associated with inflamed environments such as TNF- α, Eotaxin, MIP-1α, and MIP-1β and IL-9 (74, 75, 81, 82, 83) are upregulated in healthy aging as well (Figure 5C,D). Therefore, there is some overlap between the two groups with regards to aging and the cytokine signatures that covary along with it. One surprising observation is that there are more VIP cytokines for healthy aging than there are for diseased aging, with 11 VIP cytokines for the control group, and only 5 for the diseased group.

**Figure 6.**
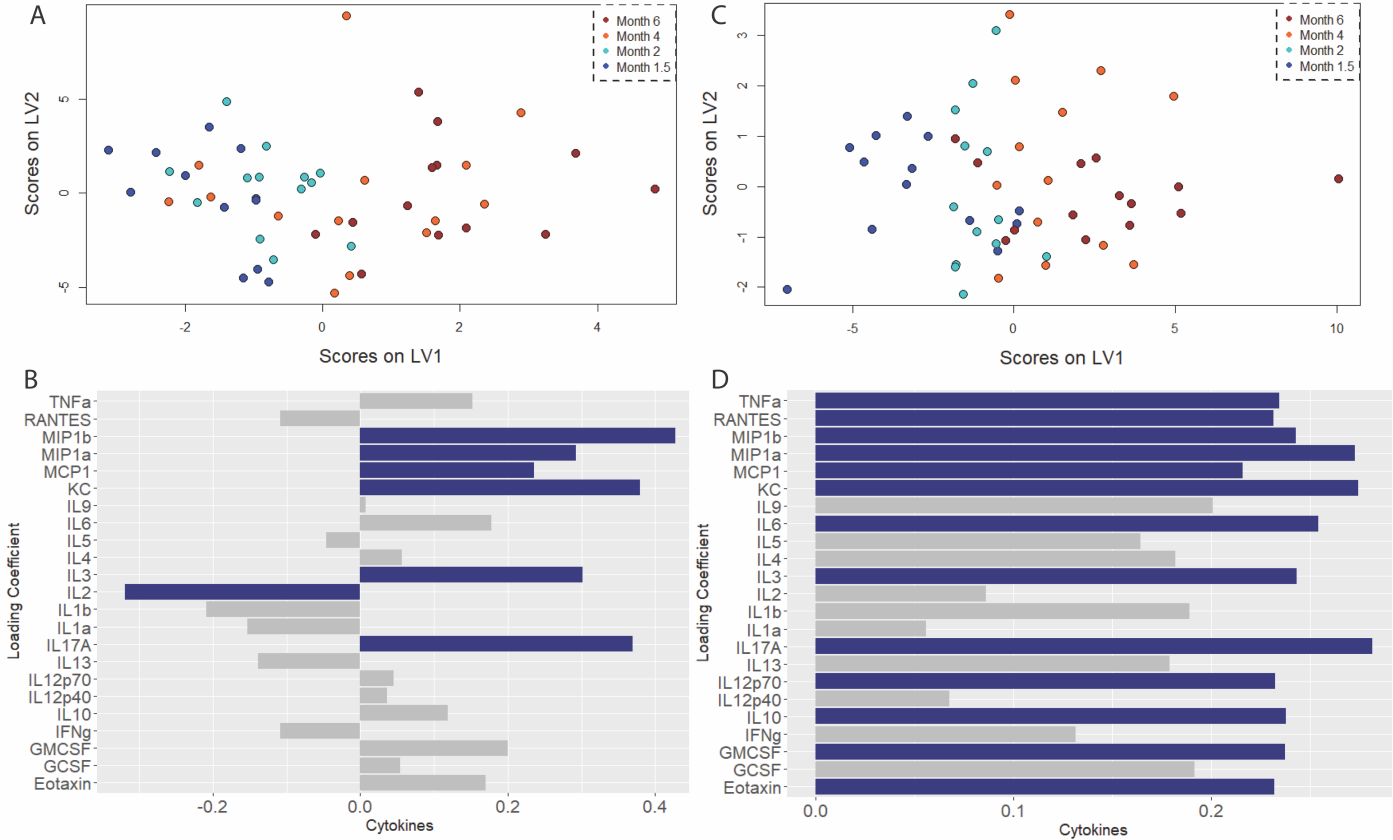
Temporal cortex exhibits progressive and specific up-regulation of microglial activation signature coinciding with onset of cognitive deficit. A) Scores plot and B) loadings on the first latent variable of an orthogonalized partial least squares regression model of cytokine levels in the temporal cortex regressed against age as a measurement of disease progression. C) Scores plot and D) loadings plot for the companion model in wild-type littermates. Colored bars indicate cytokines with VIP score > 1.

Other brain regions such as the Frontal Cortex displayed a different cytokine signature, but similar pattern, that covaried with age. We note that IL-17A (or also known as IL-17), CXCL-1, and MIP-1β are upregulated in disease aging/disease progression (disease model had 2 LVs, p = 2.120 e-2, RMSECV = 30.391, Figure 6A). We also observe that IL-2, a cytokine that has shown to have a regulatory role in inflammation and neuronal activation (85–87), is downregulated in the diseased brain (Figure 6B). RANTES is also seen to be downregulated in diseased aging, and interestingly enough there is literature demonstrating that RANTES and IL-2 may have a synergistic relationship to promote an ‘optimal’ inflammatory response (88, 89), thus suggesting that the downregulation of the two in the diseased group, but elevation of RANTES and a no-factor effect of IL-2 in healthy aging suggests that the two may form a neuroprotective role within the context of the 5xFAD disease development timeline. In contrast to the diseased group, healthy aging of the Frontal Cortex involves significantly more cytokines (as determined by the VIP score, and their relative loading weight to the disease group) with 13 cytokines being labeled as covariates that drove the variance in healthy aging (1 LV, p = 3.474 e-5, RMSECV = 37.921, Figure 6C). The disease model only had 7 cytokines with the same interpretation. Thus, relative to the 5xFAD group, healthy aging has IL-10, Eotaxin, RANTES, IL12-p70 and IL-5 to be more upregulated. Interestingly enough, the profile here consists of cytokines canonically associated with inflammation such as Eotaxin and RANTES (82, 89, 90), and those that are seen as regulators of the inflammatory environment such as IL-10 and IL12-p70 (91, 92, 93). Interestingly enough, just like the observation made for healthy aging in the Parietal Cortex, the Frontal Cortex seems to have more cytokines that covary with age relative to disease progression (Figure 6D).

**Figure 7.**
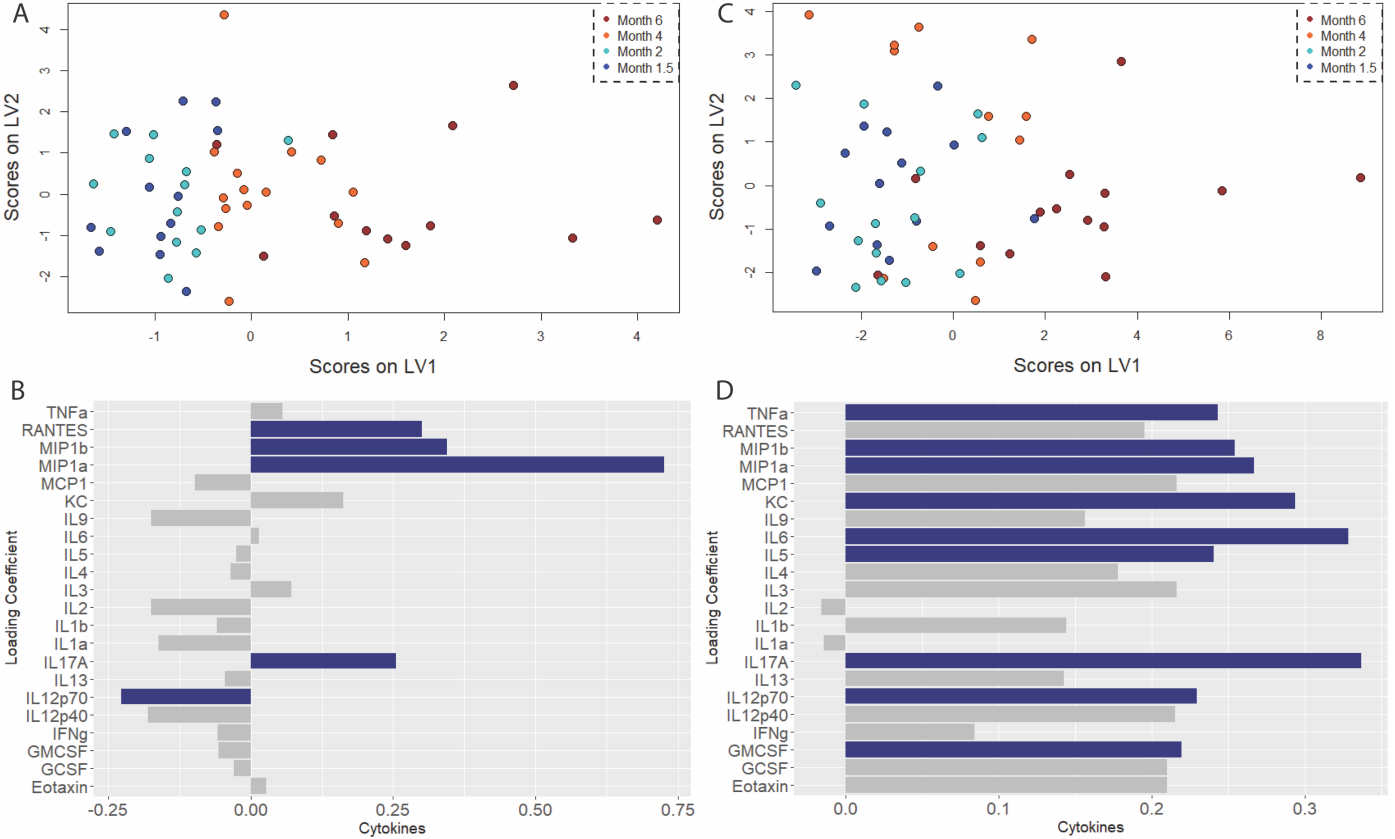
Frontal cortex exhibits progressive and specific up-regulation of microglial activation signature coinciding with onset of cognitive deficit. A) Scores plot and B) loadings on the first latent variable of an orthogonalized partial least squares regression model of cytokine levels in the frontal cortex regressed against age as a measurement of disease progression. C) Scores plot and D) loadings plot for the companion model in wild-type littermates. Colored bars indicate cytokines with VIP score > 1.

The temporal cortex also exhibits a similar pattern, but different cytokine signature, to the Frontal and Parietal Cortex. The disease model (1 LV, p =2.995 e-3, RMSECV = 30.238, Figure 7A) is shown to have RANTES, MIP-1α, MIP-1β, and IL-17A to be upregulated, While having IL12p70 to be down regulated (Figure 7B). By comparison, the healthy aging model (2 LVs, p = 7.074 e-6, RMSECV = 35.251, Figure 7C) has a myriad of cytokines that are upregulated and covary in the same direction as aging. Notable ones would be CCL1 (KC), IL5, IL6, IL12p70, IL17A, and interestingly enough TNF-α (Figure 7D). Thus, the temporal cortex cytokine profile for healthy aging is a mixture of both inflammatory and anti-inflammatory proteins (73, 82, 89, 90–94), a mixture that is distinct when comparing it to the other two cortical regions. The VIP cytokines in the disease model have common features to the cytokines profiles of the Frontal and Parietal cortex, with the commonality being MIP-1α, MIP-1β, and IL17A all being upregulated, and are classified as important covariates, with relation to disease development. Just like the healthy aging models for the Parietal and Frontal Cortex, the Temporal Cortex healthy aging model has significantly more cytokines that have their expression in the brain region positively covary with age, with 9 cytokines being VIP in the healthy aging model, and only 5 in the disease model.

## Discussion

The understanding of Alzheimer’s Disease and its etiology is still incomplete, and the consequence of this is the impedance of therapeutic strategies that could successfully reverse AD pathology (70). As such we have sought to expand the existing understanding on how the progression of the neurodegenerative disease afflicts cognitive networks as the ultimate objective would be to ameliorate the impairments that occur over the development of Alzheimer’s Disease. The reason for this is because Alzheimer’s proteinpathy does not necessitate mortality, and instead it leads to other conditions that increase the risk of mortality such as the inability to take care of one’s self, increased risky behavior (97), and the inability of basic physical functions such as the ability to swallow ingested food, leading to pneumonia (95, 96). Therefore, we have executed a comprehensive, multi-modal experiment to quantify two features of Alzheimer’s Disease; the changes in cognitive networks over disease progression, and chronic neuroinflammation in the presence of AD pathology (64, 102–105).

There is extensive literature demonstrating how functional networks are altered between fully symptomatic AD patients and their respective healthy controls (98–101). However, one key feature that is missing is how functional networks are modulated over the progression of AD, excluding information on how and when the global network may be losing the ability to have segregated, and specialized networks that could contribute to executing complex cognitive tasks (106–108). To fill this gap in our understanding of AD development, we have used a well stereotyped mouse model of AD and quantified its respective network metrics at 4 distinct time points (Figure 2) that correlate to disease states (42–46, 109). Our findings show that early disease pathology at months 1.5 and 2 exhibit profound changes to networks involving the thalamus and striatum/pallidum. Both the anatomical system and the individual ROIs within the Striatum/Pallidum, and Thalamus exhibited significant differences between the disease and healthy aging group. This is of particular interest given the roles of these two brain regions regarding cognitive functions. Firstly, the striatum and pallidum are part of the basal ganglia, a region that has the primary role of regulating motor control, and a role in higher order cognitive functions such as emotional processing and reward behavior (111–113). The thalamus, on the other hand is highly integrated within the central nervous system. Thus, it is commonly interpreted that the Thalamus plays a major role in multiple cognitive functions. For example, the Thalamus receives neuromodulatory inputs and excitatory inputs from a myriad of brain regions, including the basal ganglia (114–119, 122), and the lesion of the Thalamus has resulted in cognitive impairments such as executive dysfunction (120), and attention deficits (121). Research into other forms of cognitive impairments such as the classical Mild Cognitive Impairment stage (MCI) reinforce the importance of the Basal Ganglia and the Thalamus in cognitive health. It has been demonstrated that Basal Ganglia dysfunction is correlated with lower metrics of cognitive health (120, 121, 123–125). Thalamic hyper connectivity with cortical regions is associated with cognitive defects in schizophrenic subjects (126, 127). Therefore, given the highly integrated nature of the Thalamus and Basal Ganglia in cognitive functions, it is of particular interest to note that our results indicate changes to Thalamic and Basal Ganglia ROIs functional connectivity at the earliest stage of disease development. This would suggest that these brain regions are afflicted early at the disease stage. One potential reason for this would be that both regions feature efferent connections from the cortex (114, 122, 126). This feature is important since intraneuronal amyloid beta accumulates in the cortex of 5xFAD mice at months 1.5 (43), leading to a potential spillover to brain regions that have bijective connections to the afflicted cortical regions; like the Thalamus and Basal Ganglia.

Another finding of our experiment is that intra-hippocampal connectivity is depressed relative to healthy aging. Both individual ROIs and at the anatomical system level exhibit the depressed connectivity characteristic. This is in line with current literature as the hippocampus is the predominant brain region known to be afflicted in Alzheimer’s Disease (43, 64, 129–132). What our results do provide new insight into is how inter hippocampal connectivity to the thalamus and striatum are impacted in the prodromal stage of disease development. Therefore, our findings are in line with existing literature detailing how there are return projections from the thalamus to the hippocampus (147), and that the two may have a synergistic role in the formation of memories (148). Additionally, it has been shown that the dorsal lateral striatum and the hippocampus may both have roles in information storage (149, 150).

Furthermore, our results provide insight as to how the global network architecture is modulated at different disease stages. We observe that the fully symptomatic disease state exhibits a lower degree of network integration and segregation relative to the healthy aging group. As the ability to segregate into specialized nodes corresponds to the ability to execute cognitive tasks (106–108), it is of extreme interest to observe how both aging and disease aging both have decreased levels of network segregation, and that the disease effect is more profound in decreasing the network’s level of segregation and integration. Similar results have been observed in human studies as well, where heightened disease pathology is associated with lower levels of functional segregation (60, 101, 133). The same trend for network integration was also observed in our results, with network efficiency decreasing in both disease and healthy aging, and that the fully symptomatic timepoint resulted in a significant difference in network efficiency. Studies that have explored network integration over the course of aging have found that expanded cognitive ability coincides with higher levels of network integration, and that aging itself is correlated with decreased cognitive functionality and levels of integration and segregation (134, 135). The interesting observation from our findings is that it is only at the terminal timepoint do these differences in network metrics exist between healthy and diseased aging. None of the metrics we’ve quantified demonstrate an appreciable difference at month 4, also known as the mid-late stage disease development. However, it is between the late stage to fully developed pathological state do the differences between healthy aging and AD progression arise. The fact that the differences in network characteristics manifest so late in disease development isn’t a total surprise given that age is one of the major risk factors for developing sporadic AD. Our results reflect similarities in these global metrics in aging, but the difference at the terminal time point could be explained by how healthy aging brains may be able to compensate for age-related functionality declines (136, 137). It is extremely likely that the diseased brain is unable to undertake such processes given the breadth of tissue damage as a result of chronic inflammation due to disease pathology (43, 64, 86, 129–132, 138).

Lastly, our findings show distinct cytokine profiles for different brain regions: Temporal Cortex, Parietal Cortex, and the Frontal Cortex. Some of cytokines expressed in their unique signatures for diseased aging are commonly found in AD contexts such as MIP-1α, and MIP-1β (139, 140). However other key cytokines typically associated with AD such as IFN-γ, TNF-α, IL-1 (141) were not found in our measured signatures. That is not to say that the listed cytokines did not exist, but that these cytokines did not covary significantly with respect to time. While the different disease models share common cytokines, they also feature cytokines unique to their own respective models. MCP-1 is upregulated in the Parietal Cortex only, While IL-2 is downregulated in the Frontal Cortex, and IL12-p70 is downregulated for the Temporal Cortex. These small differences demonstrate the different aspects between the regional disease models. MCP-1 is a chemokine expressed by microglia (143), and knocking out MCP-1 from transgenic mice has been correlated to making the animals resistant to neurodegeneration (144). However, the downregulation of IL-2 and IL12-p70 in the other two cortices suggest that disease progression may progress differently in those brain regions relative to the Parietal Cortex. The reason for this hypothetical is because IL-2 and IL12-p70 are canonically associated with neuroprotective roles (88, 89, 92, 93). The regional cytokine profiles of disease progression give credence to the empirical evidence that different cortical lobes experience brain atrophy at different rates over the progression of AD. It has been shown that the parietal lobe experiences greater levels of atrophy at the prodromal stages of AD, While the frontal cortex is shown to experience damage at later stages with a lower degree of atrophy (145, 146). The regional specific rate of atrophy, and the regional specific cytokine signatures from our results indicate that the varying degree of disease pathology and its corresponding consequences are mediated by the expression of neuroprotective cytokines. It is only at the later stages of disease development do the expression of IL-2 and IL12-p70 fall off, which lines up with existing literature on how the frontal lobe atrophies at a slower, and at a later timepoint relative to the Parietal cortex. Our results also elucidated healthy aging and the corresponding cytokine signature. A qualitative commonality between the brain regions is that the healthy aging cytokine profile is more comprehensive than their diseased counterparts; featuring almost twice the number of cytokines than those featured in diseased model. Even though the heathy aging models featured canonically proinflammatory cytokines such as TNF-α, MIP-1α, MIP-1β, and CCXL1 (71–75, 83), the healthy signatures also featured at least one anti-inflammatory cytokine such IL12-p70 (92, 93) for the Frontal and Temporal cortex, and IL-4 (76, 78) for the Parietal Cortex. It is possible that the upregulation of both anti and pro inflammatory cytokines may produce the desired neuroprotective effect, as shown in the synergistic relationship between IL-2 and RANTES (88, 89). We observe a similar trend regarding IL-2 and RANTES in the frontal cortex for diseased aging, where both RANTES and IL-2 are downregulated as a result of disease progression.

## Conclusion

Here, we identify regional cortical specific cytokine signatures that are predictive of disease development. We also identify regions of interest where functional circuits could be potentially implicated in the early stages of Alzheimer’s Disease. We also observe how network segregation and integration are impacted over disease development, and is in line with increasing disease pathology, in the form of neuroinflammation, in the cortical regions. All in all, we have elucidated how AD pathology can progressively impact the brain’s network architecture, and the progressive changes to the architecture are correlated with increasing levels of neuroinflammation at the cortical regions of the brain.
